# Characterization of *Desertihabitans sp.* FB5, a halophyte associated Actinomycetes producing phytohormones

**DOI:** 10.6026/973206300214065

**Published:** 2025-11-15

**Authors:** Fehmida Bibi, Muhammad Imran Naseer, Peter Natesan Pushparaj, Absarul Haque, Esam Ibraheem Azhar

**Affiliations:** 1 Special Infectious Agents Unit-BSL3, King Fahd Medical Research Centre, King Abdulaziz University, Jeddah, 21589, Saudi Arabia; 2 Institute of Genomic Medicine Sciences, King Abdulaziz University, Jeddah 21589, Saudi Arabia; 3 Department of Medical Laboratory Sciences, Faculty of Applied Medical Sciences, King Abdulaziz University, Jeddah, 21589, Saudi Arabia; 4 King Fahd Medical Research Center, King Abdulaziz University, Jeddah 21859, Saudi Arabia

**Keywords:** Halophyte, 16S rRNA gene sequence, *Desertihabitans sp.* FB5, bioactive compounds, mass spectrometry

## Abstract

Halophytes are remarkable plants that have evolved unique strategies to thrive in saline environments. Microbial communities of halophytes are being studied extensively as potential sources of bioactive compounds. Therefore, it is of interest to identify the secondary metabolites of the rhizospheric bacterial *Desertihabitans sp.* FB5 from the halophyte *Salsola Imbricata*. Strain *Desertihabitans sp.* FB5 was identified using a molecular technique (16S rDNA) and showed a similarity of 99% to Desertihabitans aurantiacus CPCC 204711T. Antifungal activity of the strain was tested against five different pathogenic fungi: *Fusarium moniliforme*, *Altenaria mali*, *Magnaporthe grisea*, *Phytophthora capsici* and *Pythium ultimum* in an *in vitro* assay. *Desertihabitans sp.* FB5 showed weak-to-moderate inhibition of different pathogenic fungi tested in the inhibitory assay. The production of lytic enzymes was evaluated using different enzymatic activities. *Desertihabitans sp.* FB5 was positive for both cellulolytic and lipolytic activities. To detect bioactive metabolites, gas chromatography-mass spectrometry (GC-MS) and liquid chromatography-mass spectrometry (LC-MS) were performed. Both analyses showed the presence of the antimicrobial compound bacitracin and phytohormones, Indole-3-acetic acid (IAA) and Gibberellin A3 (GA3) in the culture extracts of strain. Our results demonstrated that halophytes are recognised as key source of potent bacterial strains capable of producing metabolites of industrial and medical significance.

## Background:

An environment with extreme settings presents adverse life conditions to living organisms, such as extreme pH, temperature, pressure, nutrients and salt concentrations. Halophytes in salt-rich environments have developed mechanisms to survive under high-salinity conditions [1]. These plants have developed specialized adaptations to tolerate high salt concentrations, which not only benefits the plants themselves but also the microbial communities that reside within them [2]. Interestingly, bacteria associated with halophytes have been found to possess exceptional capabilities, including the production of antimicrobial compounds that could have significant implications in the field of drug discovery. These secondary metabolites present several biological activities including anticancer activity, antimicrobial activity, anti-inflammatory effects, neuroprotective effects and diverse pharmacological properties [3, 4]. Bioactive metabolites are chemical compounds produced by living organisms, including microorganisms, plants and seaweeds, that exhibit biological activity and are potential therapeutics. The Red Sea is known for its diverse seagrass species, with at least ten different species found along the Saudi Arabian coast. These seagrass communities play a crucial role in coastal ecosystems as they provide habitat and food for numerous marine organisms. In addition, research has shown that seagrasses in the Red Sea produce active metabolites, which are unique chemical compounds with potential pharmaceutical and biotechnological applications [5]. These active metabolites have been found to possess antimicrobial, antiviral, anti-inflammatory and antioxidant properties, making them valuable in the development of new drugs and treatments [6]. Furthermore, seaweed-associated bacterial communities are a rich source of active metabolites with various biological activities [6, 7]. These bioactive compounds may have potential applications in medicine, agriculture and other industries. Seaweeds in the Red Sea are of particular interest to scientists and pharmaceutical companies because of their high concentrations of bioactive compounds. Bioactive compounds in the seagrass and seaweed communities of the Red Sea underscore the importance of studying and conserving these marine ecosystems for their potential contributions to medicine and various industries. The Red Sea is a treasure trove of active metabolites and seagrass and seaweed communities harbor diverse bioactive compounds. In recent years, there has been growing interest in exploring marine bacteria as a potential source of bioactive metabolites [8, 9]. This is due to the fact that marine bacteria have been found to produce a wide range of unique and biologically active compounds. These bioactive metabolites have shown promising potential for various applications, such as pharmaceuticals, cosmeceuticals and agrochemicals. Furthermore, marine bacteria have the advantage of being able to adapt to extreme environmental conditions, which increases the likelihood of discovering novel and diverse metabolites with unique functional properties. Their ability to produce bioactive compounds makes them valuable resources for drug discovery and development. Additionally, the ability to cultivate marine bacteria in a controlled environment, such as fermenters, allows for sustainable production of these bioactive metabolites without harming the marine ecosystem. In our previous studies, many novel bioactive marine bacteria that produce bioactive metabolites have been isolated from halophytes [10, 11, 12, 13- 14]. However, little is known about the biosynthetic potential of rhizospheric actinobacteria in halophytes. Therefore, it is of interest to identify *Desertihabitans sp.* FB5 isolated from rhizospheric soil of *S. imbricata* by 16S rRNA gene, evaluate the antimicrobial and enzymatic potential and analyze the bioactive secondary metabolites from the culture extract of *Desertihabitans sp.* FB5.

## Materials and Methods:

## Sample collection and isolation of strain:

The halophyte *S. imbricata* was collected from the North Obhur region in Jeddah, Red Sea (Figure 1 - see PDF). Soil samples adhering to the roots of the plants were used for isolation. Rhizopheric bacterial strains associated with plant roots were isolated by dipping roots in filtered autoclaved sea water (FAS) and serial dilutions were made (10-3-10-5) in FAS and spread on different media as described previously [12].

## Screening of strain for antifungal activity:

Five different fungal pathogens: Potential strains were tested against plant fungal pathogens including *Fusarium moniliforme* (KCTC 6149), *Altenaria mali* (KCTC 6972), *Magnaporthe grisea*, *Pythium ultimum*, *Phytophthora capsici* obtained in this laboratory and were used in an *in vitro* assay. The antagonistic potential of the strain was tested to evaluate pathogen growth inhibition using a described previously [13]. The antagonistic activity was estimated by determining the zone of fungal growth inhibition around the bacterial streak.

## Assessment of extracellular enzymatic activity:

The hydrolytic enzymatic activities were tested using different assays. Protease assays were performed on 1/2 R2A agar plates supplemented with skim milk. Amylase production was measured in starch media. Tributyrin supplemented with 1/2 R2A agar media was used for checking lipase activity. CMC agar medium was used to detect cellulase activity. *Desertihabitans sp.* FB5 was streaked onto the respective enzyme media at 28°C for 48 h. For the assessment of cellulase activity, after 48 h, the plates were washed with 0.1% Congo red solution, shaken for 15 min on an orbital shaker and finally washed with 1M NaCl.

## Bacterial DNA extraction, 16S rRNA gene sequencing and phylogenetic analysis: 

To identify the selected strain, genomic DNA was extracted using a DNA extraction kit. Further amplification of the 16S rRNA gene fragment was performed using previously described primers and conditions [12]. PCR product was purified using a PCR purification kit (Thermo Scientific) and sequenced by Macrogen (Seoul, Korea). The selective strain, *Desertihabitans sp.* FB5 was identified by 16S rDNA gene analysis [12]. The EzTaxon server (https://www.ezbiocloud.net/) was used for blast search and identification of the strain [15]. The phylogenetic position of the strain was determined by comparing the 16S rRNA gene sequences with the type strain sequences. Phylogenetic analysis of the strains was performed using CLUSTAL [16]. Multiple alignments and the BioEdit software [17]. For editing gaps for phylogenetic tree construction, the MEGA6 program with 1000 bootstrap values in the neighbor-joining method was used [18].

## Optimization of Culture conditions:

Strain *Desertihabitans sp.* FB5 was used to detect active metabolites from the culture extract of the strain. For this purpose, culture conditions were optimized using different media. Different media (marine broth, 1/2 R2A and 1/2 TSB) with low concentrations at different incubation periods (24 h, 36 h and 72 h) were used. Temperature and pH conditions were also optimized using a temperature range of 25-40°C and pH conditions ranging from 6 to 12. Antifungal activity was checked after optimizing the culture conditions at best conditions.

## Metabolite identification using LC-MS and GC-MS:

*Desertihabitans sp.* FB5 culture grown under optimized conditions and bacterial culture grown for 36 h were placed further at -70°C for 5 min and then at 37°C. After repeating this process three times, further centrifugation was performed for 15 mins (12000-13000g). After centrifugation, acetonitrile (10 ml) was mixed with 3 ml of the supernatant and vortexed (50sec). Again, centrifugation was performed for 15 mins at 13000 g and 500µl of the supernatant was used for LC-MS analysis. The liquid chromatography conditions were the same as described previously [12] and the raw data obtained were further processed using Agilent Mass Hunter (version B.06.00) for analysis. An in-house database was used to identify metabolites in the culture extract of *Desertihabitans sp.* FB5. For GC-MS analysis, the bacterial culture (1 ml) was lysed and centrifuge at 13000 g (10 min). The supernatant was chemically treated and different steps were performed [12]. Shimadzu GCMS-QP2010 Ultra was used to analyze the samples under the conditions described previously [12].

## Statistical analysis:

After aligning the data with the Statistic Compare component, the file contained all detailed information about the peaks and metabolites. Different databases have been used to identify bacterial metabolites [12].

## Nucleotide sequence accession numbers:

Nucleotide sequences of strain *Desertihabitans sp.* FB5 was deposited in the GenBank database under accession number PQ303222.

## Results:

## Isolation and screening of bacteria against the fungal pathogen:

*Desertihabitans sp.* FB5 was isolated from soil-adhering root samples of the halophyte *S. imbricata*. Different types of media were used for the isolation of rhizospheric bacteria and this strain was recovered from 1/2 R2A media in seawater. This strain was further tested for antifungal potential against the fungal pathogens mentioned above. Strain *Desertihabitans sp.* FB5 showed strong inhibition with 5-6 mm against *M. grisea* and *P. capsica* while weak inhibitory activity (2-3mm) was observed against *F. moniliforme* and *Py. Ultimum*. Strain *Desertihabitans sp.* FB5 exhibited no inhibitory activity against *A. mali* (Table 1 - see PDF).

## Production of extracellular enzymes:

The presence of different enzymes was tested, as described above. Strain *Desertihabitans sp.* FB5 was positive for lipase and cellulase production, but negative for protease and amylase production.

## Taxonomic and phylogenetic analysis of strain:

Strain *Desertihabitans sp.* FB5 was identified using the 16S rRNA gene sequence. This strain had 99% similarity based on the 16S rRNA gene sequence with the related type strain Desertihabitans aurantiacus CPCC 204711T from the family Propionibacteriaceae from class Actinomycetia. The phylogenetic relationships of the strains were determined based on 16S rRNA gene sequences. A neighbor-joining (NJ) phylogenetic tree was generated using the 16S rRNA gene data of the strain *Desertihabitans sp.* FB5 and closely related type strains of the genus Desertihabitans and related genera. In the phylogenetic tree, high bootstrap values were noted and branching patterns remained even (Figure 2 - see PDF). This strain formed a separate clade with related type strains of the genus Desertihabitans, with a high bootstrap value of 95%. Different clades with high bootstrap values (60-100%) were identified for the related genera of the class Actinomycetia.

## Optimization of culture conditions and identification of metabolites: 

Strain *Desertihabitans sp.* FB5 was analyzed to identify secondary metabolites from the culture extract. After testing different media and culturing conditions, the strain showed maximum inhibitory activity against the tested pathogens in modified 1/2 R2A broths at 29°C and pH 7 after 48 h of incubation. Both GC and LC-MS analyses were used to identify metabolites from the culture extract. Strain *Desertihabitans sp.* FB5 appears to be a potential strain capable of producing different metabolites, including potent bioactive compounds (Figure 3 - see PDF). LC-MS analysis confirmed the presence of a secondary bioactive compound in positive mode (Figure 3 - see PDF). This compound includes bacitracin, an antibiotic mainly used in infections. In GC-MS, peaks of 264 compounds were identified, with only a few showing the presence of the plant growth hormones indolelactic acid and Gibberellin A3 (Figure 4 - see PDF). The details of all the bioactive compounds detected by both analyses are provided in Table 2 (see PDF).

## Discussion:

Halophytes, a unique group of plants adapted to thrive in saline environments, have evolved to grow under extreme conditions, a subject of interest for scientists and researchers. They have a facultative interaction with associated bacteria that beneficially affects the growth and development of their hosts, promoting physiological adaptation responses under stress caused by salinity. Recent studies have revealed the important role of rhizobacteria in the survival and growth of hardy plants [19]. The rhizosphere, the zone surrounding plant roots, is a dynamic and complex ecosystem that teemes with a diverse array of microorganisms, including bacteria, fungi and archaea. They are considered an asset from the standpoint of bioactive molecules and fine chemicals and are useful in clinics, pharmaceuticals, biotechnological applications and agriculture. Various bioactive molecules are encapsulated within this large group of bacterial secondary metabolites, such as antibacterial, enzymatic, biosurfactant, antivirulence and immune response modulators [12, 20]. The halophyte *S. imbricata* has been widely studied for various medical and biological activities, including anti-inflammatory, antidiabetic, oral contraceptive, diuretic, antioxidant and central nervous system (CNS) depressant activities. Previous phytochemical investigations of this plant resulted in the isolation of biphenylpropanoids, triterpene saponins, flavonoid glycosides, coumarin glycosides and phenolic compounds [21]. These classes of chemical compounds and many other previously reported compounds from this plant, only our study is available regarding the identification of bioactive compounds from potential antagonistic strains [12]. Chemical investigation of the culture extract of *Desertihabitans sp.* FB5 resulted in the identification of different compounds, including bioactive molecules, from a new strain of Desertihabitans. *S. imbricata* has medicinal and biological activities, but also harbours bacterial communities as evident from our previous study [12]. A wide variety of microorganisms, including rhizobacteria, which stimulate plant growth, can be found in the rhizosphere [22]. These beneficial bacteria are remarkably effective at mitigating the detrimental effects of salinity on plant physiology, which improves the overall performance of halophytes in salinized environments. Using a variety of strategies, rhizobacteria help halophytes overcome the osmotic and ionic stressors caused by high salinity levels, allowing these plants to flourish in environments that would otherwise be hostile to them [23]. Rhizobacteria can directly exert biocontrol actions through the production of several antimicrobial substances such as lipopeptides, polyketides and phenazines to kill plant pathogens. In the present study, the rhizosphere strain *Desertihabitans sp.* FB5 is a potentially antagonistic strain. This strain showed strong antifungal activity against *M. grisea* and *P. capsica*, *F. moniliforme* and *Py. Ultimum*. Identification of antimicrobial activity of *Desertihabitans sp.* FB5 belongs to the order Actinomycetes, supporting previous studies showing that actinomycetes are a valuable source of bioactive metabolites [24].

This is first report of an antagonistic strain from genus Desertihabitans as other two strains were not checked and identified for antimicrobial activity. The presence of enzymatic activity is also consistent with previous studies, in which species from Actinomycetes were reported to produce a wide array of enzymes. Several enzymes, including protease, amylase, lipase, pectinase, cellulase and xylanase, are produced by actinomycetes [25]. Halophiles are a possible source of novel enzymes, including proteases, amylases, xylanases, lipases and gelatinases, which work under salt stress. In saline environments, certain enzymes synthesized by halophiles can be beneficial for bioremediation of contaminants. R2A broth, which is used as a culture medium under ideal culture conditions, increased the production of bioactive metabolites. Maximum antagonistic activity was observed in modified 1/2 R2A broths at 29°C and pH 7 for 48 hrs of growth culture. Most polar metabolites, particularly those containing phosphate, are the focus of LC-MS and cannot be examined with GC-MS. The presence of different chemical compounds that have been previously identified for their bioactivity but are not novel, was confirmed by LC-MS. LC-MS analysis confirmed the presence of a few secondary bioactive compounds including Bacitracin. Bacitracin is a type of antibiotic that works by inhibiting the growth of bacteria, particularly Gram-positive bacteria, by interfering with the synthesis of the bacterial cell wall [26]. Bacillus licheniformis and Bacillus subtilis are the primary producers of bacitracin, a significant natural antibacterial substance with a broad antimicrobial spectrum, high activity and poor resistance. It prevents germ growth by preventing the formation of bacterial cell walls. Detection of Bacitracin from the culture extract of *Desertihabitans sp.* FB5 was the first report of its detection in a strain of the genus Desertihabitans associated with a halophyte. Recently, a strain of Bacillus paralicheniformis NNS4-3 isolated from a mangrove plant was reported to produce potent bacitracin-like compounds [27]. Marine bacteria are stimulated to generate chemicals that differ from those found in terrestrial equivalents under complex and high-stress marine conditions, including temperature, pressure, oxygen concentrations, radiation exposure and salt concentrations. Owing to their distinct chemical modes of action, some peptides isolated from marine environments have shown promise as therapeutic candidates. Plant growth hormones such as Gibberellin A3 and Indole-3-acetic acid were identified using GC-MS. Beneficial bacteria that live in the rhizosphere and have the unusual capacity to generate, release and regulate phytohormones such auxins, cytokinins, gibberellins and jasmonic acid (JA) are known as phytohormone-producing rhizobacteria. Gibberellins play several roles in promoting seed germination and breaking seed dormancy while Indole-3-acetic acid producers use a variety of strategies to encourage plant growth [28]. Based on the results of LC-MS and GC-MS analyses, the bioactive molecules and phytohormones found in *Desertihabitans sp.* FB5 has both medical and agricultural applications.

## Conclusion:

We show that halophytes contain novel metabolites. Strain *Desertihabitans sp.* FB5 displays the spectra of bioactive substances such as well-known antibiotics and phytohormones. The Red Sea coast in Saudi Arabia is home to antagonistic bacteria from halophytes that can produce a wide variety of antimicrobial compounds that can be employed as biocontrol agents in agriculture and medicine.

## References

[1] Rozentsvet O.A (2020). Dokl Biol Sci..

[2] Zheng R (2021). Int J Mol Sci..

[3] Zheng Y (2021). Microbiol Spectr..

[4] Matulja D (2022). Molecules..

[5] https://link.springer.com/book/10.1007/978-3-319-99417-8.

[6] Singh R.P, Reddy C.R.K (2014). FEMS Microbiol Ecol..

[7] Amone-Mabuto M (2023). Ocean Coast. Manag..

[8] Rateb M.E, Abdelmohsen U.R (2021). Mar Drugs..

[9] Karthikeyan AJ (2022). Genet. Eng. & Biotechnol..

[10] Bibi F (2018). 3 Biotech..

[11] Bibi F (2017). Genet. Mol. Res..

[12] Bibi F (2018). Front microbiol..

[13] Bibi F (2018). Biocontrol Sci. Techn..

[14] Bibi F (2017). Genet. Mol. Res..

[15] Yoon S.H (2017). Int J Syst Evol Microbiol..

[16] Thompson J.D (1997). Nucleic Acids Res..

[17] Hall T.A (1999). Nucleic Acids Symp. Ser..

[18] Tamura K (2013). Mol. Biol. Evol..

[19] Kalam S (2020). Front. microbiol..

[20] Strobel G (1999). Microbiology..

[21] Suleiman R.K (2022). ACS omega..

[22] Giannelli G (2023). Plants..

[23] Bhat M.A (2020). Microbiol..

[24] Oyedoh O.P (2023). Biotechnol Adv..

[25] Salwan R, Sharma V (2020). Microbiol Res..

[26] Zhu J (2023). Synth Syst Biotechnol..

[27] Sermkaew N (2024). Antibiotics..

[28] Ozimek E (2018). Int J Mol Sci..

